# Altered Metabolites in Hepatocellular Carcinoma (HCC) Paving the Road for Metabolomics Signature and Biomarkers for Early Diagnosis of HCC

**DOI:** 10.7759/cureus.71968

**Published:** 2024-10-20

**Authors:** Hiba S Al-Amodi, Hala F Kamel

**Affiliations:** 1 Biochemistry, Umm Al-Qura University, Makkah, SAU; 2 Medical Biochemistry and Molecular Biology, Ain Shams University, Cairo, EGY

**Keywords:** acylcarnitines, carcinogenesis, hepatocellular carcinoma, metabolism, metabolomics

## Abstract

Globally, hepatocellular carcinoma (HCC) is one of the most commonly encountered cancers. Because the current early diagnostic tests for HCC are not very sensitive, most cases of the disease are discovered late when it is in its terminal stage. Cellular metabolism changes during carcinogenesis to enable cancer cells to adapt to the hypoxic milieu, boost anabolic synthesis, promote survival, and evade apoptotic death signals. Omic techniques represent a breakthrough in the field of diagnostic technology. For example, Metabolomics analysis could be used to identify these metabolite alterations. Understanding the metabolic alterations linked to HCC is crucial for improving high-risk patients' surveillance and understanding the illness's biology. This review highlights the metabolic alterations linked to energy production in cancer cells, as well as the significantly altered metabolites and pathways associated with hepatocarcinogenesis, including acylcarnitines (ACs), amino acids, proteins, lipids, carbohydrates, glucose, and lactate, which reflect the anabolic and catabolic changes occurring in these cells. Additionally, it discusses the clinical implications of recent metabolomics that may serve as potential biomarkers for early diagnosis and monitoring of the progression of HCC.

## Introduction and background

As the liver is an essential organ regulating energy and metabolism, hepatic injuries, particularly oncogenesis, can intensely disturb its metabolism [1,2]. Histopathological investigations and clinical and imaging procedures are important for the confirmation of liver diseases [3]. Aside from the time it takes to collect clinical data, one of the drawbacks of imaging examinations is that they cannot reach sensitive diagnoses [4,5]. Although liver biopsy is the gold standard procedure, it is considered an invasive diagnostic tool for hepatic disorders [6]. Because of high rates of false negative and false positive findings, standard serology tests, such as alpha-fetoprotein (AFP), are no longer employed as effective screening techniques in chronic hepatic patients [7]. Therefore, an early and non-invasive technique for liver disease is considered a great challenge for clinicians. For the discovery of specific and sensitive hepatic predictive markers, extensive efforts have been performed [8]. Non-invasive diagnostic procedures and novel techniques resolve these boundaries and can be useful in the early-stage diagnosis of liver disease as well as they could reduce the requisite for liver biopsy in hepatic patients. Together with other omics technologies, metabolomics contributes to a comprehensive knowledge of metabolic and biochemical changes within the cell and their connections with one another in the biological systems [8,9].

Metabolomics is the study of low molecular weight biomolecules within an organism or biological samples on a quantitative time scale under certain environmental circumstances. Peptides, carbohydrates, vitamins, alkaloids, polyphenols, nucleic acids, amino acids, and organic acids have been identified as metabolites with low molecular weight, <1000 Da within cells, tissues, or organisms [10]. Metabolomics analysis is a novel technology used in the discovery of biomarkers and dynamic fields, leading to a universal understanding of organ systems, similar to other omics methods, including proteomics, transcriptomics, and genomics. Metabolomics technique is an important tool to differentiate between diseased and non-diseased conditions [11]. Achieved results from metabolomics studies have proposed that profiles of metabolomics might have the potential to be used in the diagnosis of diseases and biomarker detection [12]. The perfect biomarker should be the one that can be discovered with high rates of sensitivity and specificity in biological samples in a less invasive manner, such as blood, saliva, or urine. Lately, metabolomics profiling approaches have been progressively used to clarify significant alterations in tumor metabolism also known as tumor metabolome, and discover candidate “biomarkers” from such variance within a large number of endogenous metabolites. Blood and urine samples are the most commonly utilized specimens for discovering the systematic change in human metabolome [13]. In comparison with blood samples, urine sample utilization is favored because it allows non-invasive monitoring of metabolomics alterations. Generally, mass spectrometry (MS)-based techniques, such as gas and liquid chromatography, as well as Fourier transform infrared (FT-IR) and nuclear magnetic resonance (NMR), are ideal methods used in metabolomics analysis [14].

A brief description and interpretation of metabolomics signatures and their accomplishments in the field of biomarkers’ identification in hepatic diseases, such as liver cirrhosis and hepatocellular carcinoma (HCC), are provided in the current review.

## Review

Hepatocellular carcinoma

Two million fatalities per year are caused by liver disease, which also accounts for 4% of all deaths globally. Liver cirrhosis, HCC, and its consequences are mostly responsible for all these deaths [15]. 

HCC is the most common kind of primary liver cancer and is ranked fifth among all types of cancers globally, with around 50000 new cases annually [16].

In addition, according to the Global Cancer Observatory, HCC represents the seventh most common cancer among males and ninth among females for all cancer cases in Saudi Arabia [17,18]. HCC has a very poor prognosis, which depends on the stage of the tumor [19]. Early diagnosis of the tumor can be achieved through the surveillance of at-risk patients using several methods, such as ultrasound (US) [13].

Bacteria, viruses, alcohol, therapeutic drugs, and harmful substances are the main pathogenic factors of HCC [20]. The primary causes of HCC occurrences are chronic liver diseases (CLD) and chronic hepatitis caused by either hepatitis B virus (HBV) or hepatitis C virus (HCV) infections [21]. Its development occurs over the long term and in a multi-step manner under the control of several genetic interactions and interplay [19]. A persisting inflammatory milieu might cause simple liver steatosis to progress into fibrosis, cirrhosis, and finally HCC [1]. Recently, the risks for the development of HCC on top of viral hepatitis have been increased, this might be due to the higher frequency of occurrence of metabolic dysfunction-associated steatotic liver disease (MASLD), which is recently defined as steatotic liver disease (SLD) in the presence of one or more cardiometabolic risk factor(s) and the absence of harmful alcohol intake [22]. Furthermore, the emergence of type II diabetes, metabolic syndrome, and obesity are major contributing metabolic factors associated with the significantly rising rate of HCC, through several oncogenic mechanisms [23,24].

The HCC research is now focusing on the pathophysiology of the disease and the development of highly sensitive and specific diagnostic and prognostic biomarkers in order to improve HCC patient outcomes [25]. The high-throughput technology, especially multi-omics techniques such as genomics, proteomics, and metabolomics, has significantly expedited advancements in HCC research [2,26,27]. Therefore, numerous sensitive and specific biomarkers have been discovered lately for the rapid and precise diagnosis of HCC [28]. As a result, monitoring the fluctuation of metabolites in a particular metabolic pathway using quantitative and qualitative techniques in HCC specimens might provide some crucial data for the pathogenesis and diagnosis of HCC [25]. Targeted and untargeted metabolomics research on HCC has recently received a lot of attention and experienced tremendous growth [29-31]. However, no single method could completely address all metabolites due to the wide variety of metabolic pathways [32].

Alterations of acylcarnitines

**Figure 1:**
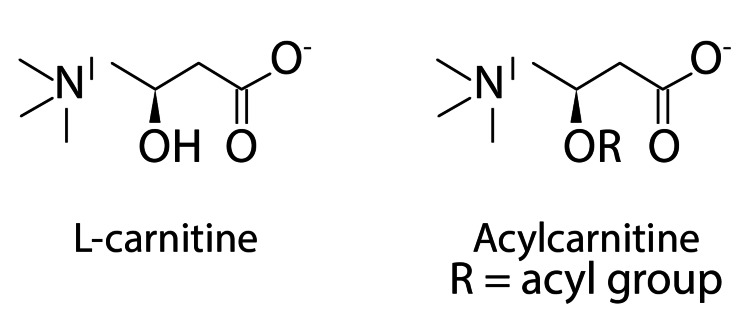
The composition of L-carnitine and ACs AC, acylcarnitine Image formed by Adobe Illustrator graphic design software (Adobe Systems Inc., San Jose, CA, USA).

Acylcarnitines (ACs) are esters arising from the conjugation of L-carnitine with an acyl group of fatty acids (FAs) (Figure 1). The broad class of ACs belongs to the non-protein amino acid family. The human Metabolome Database claims that the human body may contain more than 1200 FAs [2,33]. Consequently, on the basis of these FAs, it is suggested that a huge number of ACs might be created. ACs are classified similarly to FAs depending on the length of the acyl groups as short-, medium-, and long-chain ACs (merely abbreviated as SCACs, MCACs, and LCACs). The zwitterionic molecules called ACs have a carboxyl group and a quaternary ammonium group as part of their structure (Figure 1) [2].

**Figure 2:**
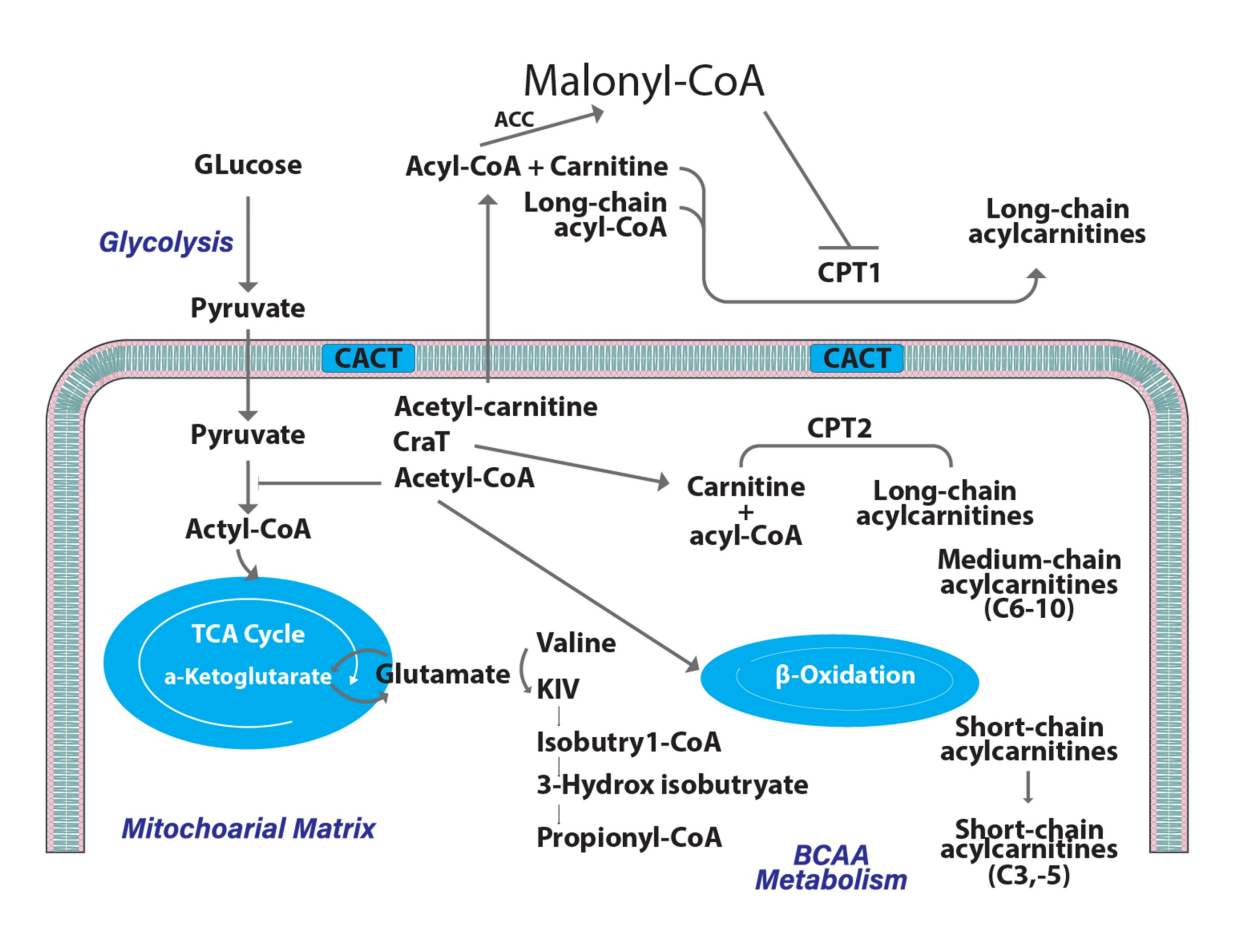
Role of AC in beta-oxidation of FAs within the mitochondria AC, acylcarnitine; FA, fatty acid Image formed by Adobe Illustrator graphic design software (Adobe Systems Inc., San Jose, CA, USA).

ACs play an essential role in cellular metabolic and physiological activities due to their large number and special structure [2,34]. ACs' primary role is in the process of oxidation of long-chain fatty acids (LCFAs) (Figure 2). They act as transporters for the active form of LCFAs into the mitochondrial matrix prior to beta-oxidation, which provides energy for a wide range of cellular processes [35,36]. The long-chain acylcoenzyme A synthetase (LACS), carnitine/acylcarnitine translocase (CACT), and carnitine palmitoyltransferases 1 and 2 (CPT1 and CPT2) are the main regulatory enzymes for these activities [2,37]. The LCACs are transported into the mitochondrial matrix through the mitochondrial membranes while being catalyzed by CACT [2,38]. Long-chain acyl-CoAs are synthesized when the LCFAs bind to coenzyme A (CoA) via the LACS activity. The outer mitochondrial membrane protein CPT1 catalyzes the conversion of the intermediates into LCACs [39]. Then, in the presence of CPT2, LCACs are transformed again into the equivalent long-chain acyl-CoAs for β-oxidation [40]. The enzyme carnitine acetyltransferase (CrAT) transforms the final products, acetyl-CoAs, into acetylcarnitines. Finally, CACT exports acetylcarnitines from the mitochondrion to the cytoplasm [36,41].

A crucial element governing the equilibrium of intracellular sugar and lipid metabolism is the ACs [2]. Additionally, they are implicated in the branched-chain amino acid metabolization [42]. They are also important in preserving the mitochondrial acyl-CoA/CoA ratio's equilibrium [2]. Lowering the glucagon/insulin ratio triggers the pyruvate dehydrogenase activity that boosts aerobic glycolysis and pyruvate oxidation [43]. By inhibiting the CPT1 activity and reducing the FA oxidation, acetylcarnitine can be transformed into malonyl-CoA in the cytosol, which eliminates the negative effects brought due to the mitochondrial buildup of acyl-CoA metabolic intermediates [44]. The physiological processes of FA peroxidation and ketone body synthesis are additionally implicated by ACs [36,45]. As a result, the metabolism of ACs is crucial for controlling the harmony between cellular sugar and lipid metabolic pathways as well as FA transport (Figure 2) [36,42]. Thus, numerous metabolic disorders might be intimately linked with ACs [2].

**Figure 3:**
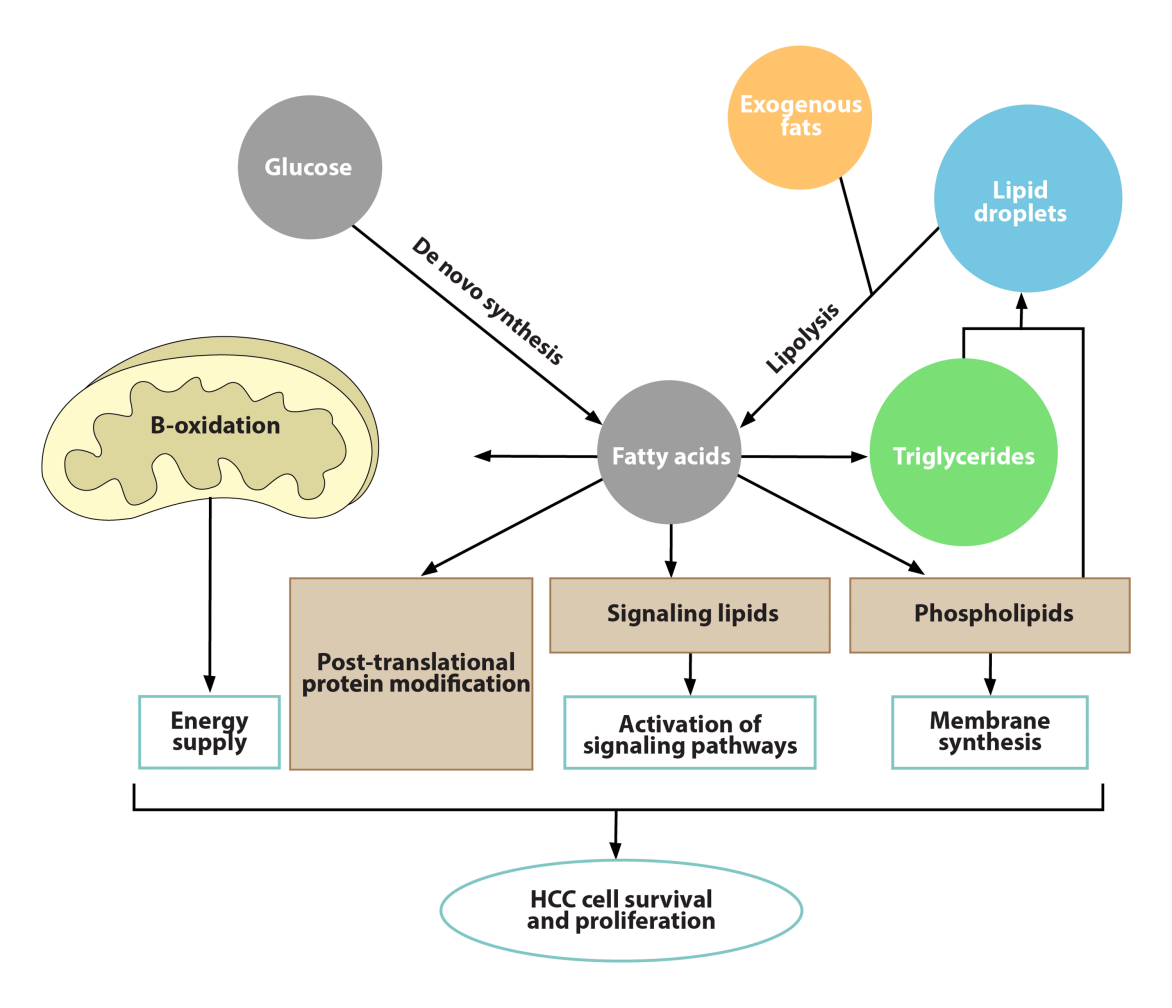
Alteration of metabolism in HCC HCC, hepatocellular carcinoma Image formed by Adobe Illustrator graphic design software (Adobe Systems Inc., San Jose, CA, USA).

For endogenous carnitine production and metabolism, the liver is the most active organ [2,46]. As a result, any diseases of the liver involving its synthetic and metabolic functions might significantly affect the ACs’ levels (Figure 3) [47]. Hepatocytes are activated at various phases of liver disease by several risk variables; hence, the glucose and lipid requirements may vary at each phase [48]. Accordingly, liver disease progression and stages are associated with alterations in AC metabolism, which generally worsen as the liver disease advances [2]. It has been suggested that butyrylcarnitine levels appeared to be markedly increased in patients with NAFLD. Free carnitine, propionylcarnitine, butyrylcarnitine, and 2-methylbutyrylcarnitine increased significantly as the condition worsened and progressed into NASH [49]. Both C16:1-AC and C18:1-AC were found to be high in cases with liver fibrosis and cirrhosis, designating a decrease of beta-oxidation for those FAs [36,50]. Diverse pathogenic causes lead to distinct alterations in AC levels. For instance, serum LCAC concentrations exhibited a rising tendency in cirrhosis patients brought on by HCV and HBV; however, both LCACs and SCACs displayed a rising tendency in cirrhosis patients complicating alcoholic intake [2,51]. Along with endogenous ACs, carnitine supplementation may potentially have an impact on the development and progression of HCC; it was found that L-carnitine-treated mice developed fewer liver tumors, which might be due to over-expression of the genes related to the transport of LCFA, oxidative phosphorylation, and anti-oxidants within the liver [52]. A screening model using mass spectrometry for differentiation between cirrhosis and HCC proposed that SCACs are risk factors for the development of HCC; on the other hand, LCACs are risk factors for cirrhosis in addition to a complete profile of amino acids and calculated ratios with ACs [53]. The ratios of AC metabolites have been studied in HCC; an increase in the ratio of acetylcarnitine to free carnitine has been reported in HCC, indicating the upregulation of a number of FAs. However, the ratio of propionyl-carnitine to free carnitine was downregulated [54].

Obviously, HCC can greatly control the pattern of ACs’ metabolism and utilization, thus a specific pattern of altered serum AC levels might be seen in patients with HCC. They were found to have elevated levels of free carnitine and LCACs, as well as reduced concentrations of SCACs and MCACs [55,56]. The SCAC and MCAC act primarily to excrete organic acids into the urine and bile after removing them from mitochondria and other organelles. The reduction of SCAC and MCAC levels indicates either a rise in organelle’s excretion rates or a barrier decreasing their accumulation rates. In contrast, the increased synthesis and transportation of LCACs into the mitochondria was accompanied by increased beta-oxidation and consequently increased energy production [57]. Similarly, several metabolomic studies have demonstrated the high levels of LCACs and low levels of MCACs and SCACs in the serum and/or cancerous tissues of HCC patients [54,55,58-60]. In spite of these consistent findings in the tissues of HCC patients in comparison to the non-tumorous side tissues, a minimal association among MCACs, SCACs, and LCACs was reported [61]. In fact, SCACs and MCACs can pass freely across the mitochondrial membrane; however, LCACs rely specifically on the carnitine shuttling system via both CPT1 and CPT2. Thus, the downregulation of carnitine shuttle regulatory enzymes such as CPT1 has been linked to the occurrence of HCC and metastasizing progression [62,63]. Additionally, the expression changes appeared to be related to the tumor size, histological grade, intrahepatic metastasis, and tumor-node-metastasis stage, which are risk indicators for the prognosis of HCC patients [64]. In the same context, If CPT2 is downregulated, this will cause a pronounced inhibitory effect on the shuttling mechanisms and accumulation of LCACs; meanwhile, SCACs and MCACs could pass freely across the mitochondrial membrane and proceed for oxidation, thus their levels are decreased [61]. These encouraging results presented mechanistic insights into AC accumulation in HCC. Due to the particular importance of AC metabolism for generating energy in HCC cases, focusing on this process is advised to be a possible procedure for carcinoma therapy [2].

Alterations in amino acid and protein metabolism in HCC

Amino acids are as important fuels for cancer growth as glucose, which is a main energy source for cancer development. For instance, glutamine, which supports the tricarboxylic acid (TCA) cycle, is mostly anaplerotic and releases both amine groups [65,66]. Further amino acids can serve as fortuitous cellular energy sources in addition to glutamine. Alternative organic molecule sources, such as branched-chain amino acids (BCAAs), valine, leucine, and isoleucine, can also power the TCA cycle [67]. Similar to bioenergetic pathways, biosynthetic pathways depend on a variety of amino acid donors. Catabolism of BCAAs can act as a substitute for acetyl-CoA production in the process of lipid synthesis [66,67]. Nucleotide biosynthesis, which can be divided into purine and pyrimidine synthesis, is another process that depends on amino acids [68]. For the purpose of purine biosynthesis, glycine, glutamine, and aspartate are suppliers of carbon and nitrogen atoms [66,69]. Glycine, serine, and methionine are additional carbon sources for nucleobases in the form of formic acid, providing one-carbon units via the methionine-folate cycle [70,71]. Aside from that, amino acids generate compounds that promote the development and spread of cancer [66]. Reactive oxygen species (ROS) built up by cancer cell proliferation can damage macromolecules and finally trigger apoptosis. Cancer cells rely on glutathione production from glutamate glycine and cysteine to promote redox balance in order to combat cancer growth. While it is common knowledge that glycolysis and the hexose monophosphate shunt (HMS) are the primary sources of NADPH for maintaining redox equilibrium, the powered folate cycle using one-carbon units produced from serine also contributes significantly to NADPH synthesis [72]. Aside from their direct involvement in reprogramming metabolism, amino acids and their derivatives are similarly crucial in facilitating post-transcriptional modification and epigenetic control. For instance, the methionine cycle's balanced metabolite levels, which are affected by glycine, serine, and methionine, regulate DNA and histone methylation [73]. Additionally, acetyl-CoA produced from amino acids is important for protein acetylation, which can aid in the growth of tumors [74]. One important family of enzymes for the development of cancer cells is transaminases that enable the interconversion of multifunctional amino acids from erroneous sources. The overexpression of essential amino acids (EAA) transporters, a characteristic of many malignancies, is a result of their increased demand to sustain cellular cancer development [75,76].

The metabolism of proteins is carried out by the liver in several ways. The liver produces a wide range of necessary proteins for the upkeeping of hemostasis, pressure from oncotic cells, transport of lipids, and hormones, in addition to acute phase reactions. Of these proteins, albumin is solely produced by the liver and makes up 40% of the synthesis of hepatic proteins. In addition, the liver can also produce very low-density lipoproteins (VLDL) apoB 100, and thyroid-binding globulin [77]. Compared to individuals with higher serum albumin levels, patients with lower levels have considerably larger HCC sizes, a higher frequency of thrombosis in the portal vein, more tumor multifocality, and increased levels of AFP [78].

Furthermore, the synthesized non-EAAs are essential for maintaining a variety of homeostatic processes, including gluconeogenesis. Except for BCAAs, both synthetic and dietary amino acids are either used for protein synthesis or catabolized through transamination or oxidative deamination processes. Keto acids, which are produced as a result of these reactions, can be oxidized to produce ATP as a source of energy [77]. Serum assays of many enzymes involved in these pathways, such as alanine transaminase (ALT) and aspartate transaminase (AST), are frequently used to evaluate liver damage. "Additionally, the oxidative deamination of amino acids results in the production of ammonium ions, a toxic byproduct that can be detoxified either in extrahepatic tissues through glutamine synthesis when coupled with glutamate or in the liver via the urea cycle, producing urea that then travels to the kidney, where it is excreted in urine [77].

According to the amino acid significance and the enzymes involved in their generation in the start and progression of cancer, an enhanced amino acid metabolism is typically seen in human malignancies [79,80]. The change toward a greater synthesis of amino acids is viewed because of the changed glucose metabolism that has been reported. Amino acids can be employed as precursors of glucose or as enzyme triggers in situations when aerobic glycolysis consumes more glucose [81].

HCC differs from other liver disorders in that it has an abnormal amino acid metabolism. For example, serum concentrations of alanine, glycine, serine, cysteine, methionine, aspartic acid, glutamic acid, tyrosine/phenylalanine ratio, tryptophan, and lysine were substantially higher in HCC patients than healthy controls, along with a smaller proportion of aromatic-amino acids (AAA) (tryptophan, tyrosine, and phenylalanine) to BCAA (valine, leucine, and isoleucine) [82].

Additionally, the bioavailability of amino acids supports cellular responses to hypoxia during the etiology of HCC in addition to boosting anabolic and catabolic pathways [83].

Alteration of carbohydrate metabolites in HCC

The liver's ability to produce, store, and release glucose depends on the body's requirement for it. The plasma membrane glucose transporter (GLUT), which is active after a meal, transports blood glucose to the hepatocytes. There are 14 members of the human GLUT protein family, and each has different tissue expression patterns and substrate specificities [84]. Once within the cell, glucose is initially transformed by the glycolytic pathway into pyruvate and fully oxidized via the TCA cycle and oxidative phosphorylation into the mitochondrial matrix. Alternately, it can be sent into the de novo lipogenesis (DNL) route for FA synthesis [77]. The rate-limiting HMS enzyme, glucose-6-phosphate (G6P) dehydrogenase, is employed in the hepatocytes to create reduced nicotinamide adenine dinucleotide phosphate (NADPH), which is essential for lipid synthesis and the creation of other bioactive compounds. The energy metabolism of glucose is altered in diseased conditions. Significant alterations have been seen in the flow of metabolites as well as the expression of certain transporters and enzyme isoforms [77].

**Figure 4:**
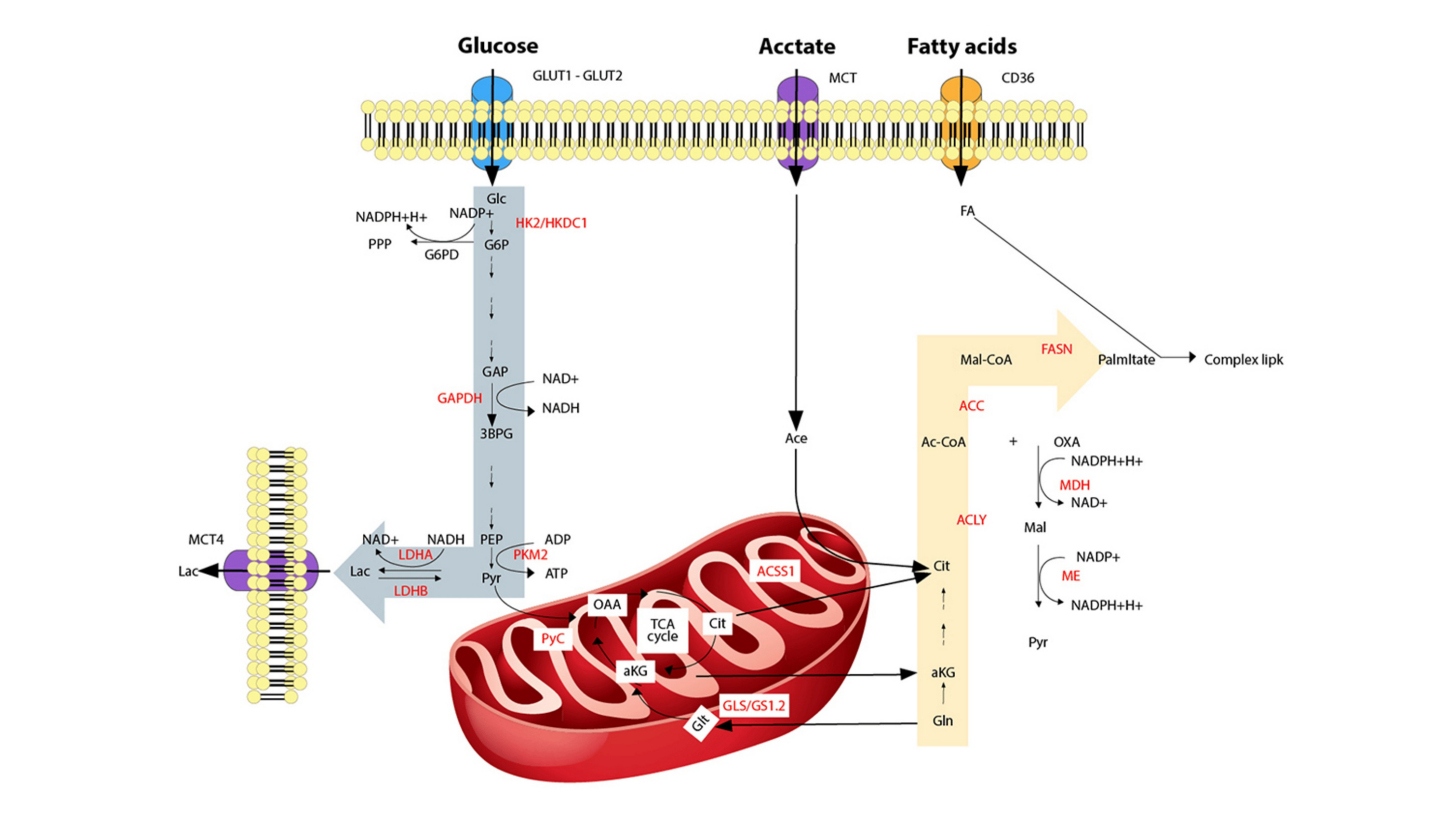
Altered glucose metabolism in HCC HCC, hepatocellular carcinoma Image formed by Adobe Illustrator graphic design software (Adobe Systems Inc., San Jose, CA, USA).

High levels of glucose metabolism are observed in HCC tumors (Figure 4) [85]. The ability of 18F-fludeoxyglucose (18F-FDG) positron emission tomography (PET) to relate to histopathologic characteristics and tumor proliferative activity provides strong evidence that this increased metabolic demand is crucial for metabolic imaging [77,86,87]. Furthermore, by producing progressive glycation end-products and O-GlcNAcylation of the yes-associated protein (YAP) and c-jun, high glucose levels like those seen in diabetic individuals might hasten carcinogenesis in HCC cells [88,89].

The Warburg effect, a characteristic of these changes, is an increase in glucose absorption, as well as lactate creation even in the existence of oxygen and completely functioning mitochondria [90]. However, this does not correspond to an increase in gluconeogenesis because HCC has been shown to have downregulated expression of fructose 1,6-bisphosphatase 1 (FBP1) and phosphoenolpyruvate carboxykinases 1 and 2 [91]. Overall, this metabolic reprogramming encourages expansion, maintenance over the long term, proliferation, and survival [77,92]. HCC tumors increase glucose absorption by upregulating GLUT1 and GLUT2 isoforms in response to this metabolic demand [93]. Hexokinase, the first enzyme in glycolysis, transforms glucose into G6P after it has entered the cell. Mammalian tissues express five major hexokinase isoforms, which are designated as HK1, HK2, HK3, HK4, and the isoform hexokinase domain containing 1 (HKDC1). The clinical stage of the tumor is associated with the expression of the HK2 isoform, which is expressed at high levels in HCC tumors [94]. Changes in many glycolytic enzymes accompany the switch to glucose metabolism. The reduced expression of FBP1 is a well-known example [95]. By changing glucose metabolism, FBP1 downregulation brought about by promoter methylation and copy-number decrease aided in the evolution of HCC [94]. Glyceraldehyde-3-phosphate dehydrogenase (GAPDH) and pyruvate kinase 2 (PKM2) were also found to express themselves more often [96,97]. The enhanced expression of these enzymes promotes the flow of glucose through the glycolytic pathway, producing pyruvate, which can then be utilized to produce lactate or sent to the TCA cycle. The first theory, which proposes an enhanced TCA carbon anaplerosis in HCC cells, is supported by both the great usage of glutamine and the elevation amounts of lactate seen in HCC tissues [94]. The major hepatic gluconeogenic substrate, glutamine, is the most common amino acid in blood and tissues. In HCC, a metabolic switch toward glutamine controls the development of the tumor [91]. A systemic impact may result from the increased metabolism and net glutamine consumption of hepatoma cells [77,98]. Glutamine is processed by a variety of unique routes. In order to refill the TCA cycle and boost energy generation, glutamine can be transformed into α-ketoglutarate by glutaminolysis. This process also produces intermediates for other metabolic pathways. To support lipid synthesis, glutamine undergoes reductive carboxylation, moving from α-ketoglutarate to citrate in the opposite direction of the TCA cycle [77]. The involvement of glutamine in HCC has been the subject of contradictory information up to this point. In reality, research showed that the predominant route by which glutamine is degraded to produce lactate is glutaminolysis, not reductive carboxylation [94].

Lactate dehydrogenase (LDH) catalyzes the metabolic process that converts pyruvate to lactate. There are five active isoenzymes of human LDH, and everyone is a tetrameric enzyme made up of the M and H (officially A and B) main subunits, which are respectively encoded by LDH-A and LDH-B. The M subunit is mostly present in the muscles of the skeletal system, while the H subunit is primarily present in the muscles of the heart. Upregulated LDH-A and LDH-B are linked to aggressive tumor outcomes in human malignancies [99]. Monocarboxylate transporters (MCT) export lactate, a byproduct of LDH action, in the extracellular environment. MCT4 overexpression has been noted in HCC samples [100]. The reprogramming of glucose metabolism in HCC cells was discovered to be mediated by basigin, a transmembrane glycoprotein also known as CD147. In particular, CD147 aided lactate export, MCT1 expression on the cell surface, and glycolysis [101].

NMR examination of HCC samples revealed an abundant amount of lactic acid and a low concentration of glucose at the metabolic level. This demonstrates the confirmation of the glycolytic transition to lactate and establishes a link between enzymatic changes and metabolite expression [102]. This metabolic shift appears to be the cause of the elevated lactate content seen in the blood of HCC patients compared to normal people [77]. The function of this released lactate is still largely unknown. However, higher levels of lactate generation were seen in NASH patients compared to healthy people, proposing that the change to anaerobic metabolism of glucose may be responsible for the early stages of hepatic cancer development [49].

Changed anabolism and catabolism in HCC patients

The liver is important for lipid metabolism, and abnormalities in these metabolic pathways may be responsible for the origination and development of HCC, as shown by the elevated risks seen in individuals with obesity, diabetes, and fatty liver disease [103-105]. Following a carbohydrate-high meal, DNL can produce FAs from glycolytic pyruvate. The pyruvate dehydrogenase enzyme converts pyruvate into acetyl-CoA as a result of mitochondria entry. Acetyl-CoA and oxaloacetate combine in the mitochondrial matrix to create citric acid, which under high energetic charge conditions is transported to the cytosol through the citrate carrier for lipogenesis. The multifunctional enzyme fatty acid synthase (FASN), which uses malonyl-CoA to synthesize palmitoyl-CoA, and acetyl-CoA carboxylase (ACC), which activates the ATP-dependent carboxylation of acetyl-CoA to malonyl-CoA, are important enzymes in cytosolic de novo lipogenesis (DNL) [106]. Additionally, DNL requires decreasing power in the form of NADPH+H+, which is mainly produced by the pentose phosphate pathway (PPP) and malic enzyme (ME) reaction during the metabolism of glucose. In HCC specimens and in other hepatic illnesses, such as NAFLD, DNL changes were seen [107,108]. Numerous DNL-related enzymes and enzymes involved in the synthesis of NADPH, namely G6PD and ME, were shown to be increased in HCC when compared to noncancerous liver samples, according to a combinatorial network-based study [91].

Excessive expression of FASN is critical for boosting the survival and development of tumor cells during cancer development and has been linked to a bad prognosis for patients [109].

Acetyl CoA may be produced using other carbon sources, which is necessary for DNL. This can come from exogenous acetate, which MCT family members transfer into cells and then use acetyl CoA synthase enzymes to convert into acetyl CoA to drive FA synthesis. When compared to noncancerous liver, HCC exhibits considerable overexpression of mitochondrial enzymes, which is linked to enhanced tumor development and aggressiveness [77,91].

Through the activation of complimentary pathways, tumors might become dependent on DNL or independent from it. In actuality, HCC tumor development might be aided by both exogenous and de novo synthesized fFAs [110]. According to their content, the liver may absorb nonesterified FAs from the blood either via diffusion or by using certain carriers (fatty acid transport protein (FATP) or FA translocase/CD36). The promotion of the epithelial-mesenchymal transition (EMT) program, a process that aids in the advancement of cancer, has been linked to the activation of the CD36 pathway and tumor aggressiveness [111].

FAs can be oxidized by the liver by both mitochondrial and peroxisomal oxidation. The activation of the enzyme CPT-I controls the FA entrance into the mitochondria [112]. CPT-I catalyzes the production of ACs from extremely long acyl-CoA and carnitine, permitting the entrance of polar FAs in the mitochondrial matrix [77]. Additionally, it has been noted that HCC patients have deregulated mitochondrial oxidation along with downregulated levels of many FA oxidizing enzymes [111]. As a result, it was shown that HCC and cirrhosis had distinct urine levels of short- and medium-chain ACs. Butyrylcarnitine (carnitine C4:0) was identified as a possible biomarker for differentiating between HCC and cirrhosis [113]. Collectively, these findings suggest that mitochondrial changes may act as an early predictor of HCC [77].

The liver is a significant site for endogenous cholesterol metabolism. The pool of cholesterol is strongly controlled and represents the dietary cholesterol intake, its synthesis, secretion, and uptake from plasma lipoproteins. The catalysis of mevalonate production by the 3-hydroxy-3-methylglutaryl-CoA reductase is the rate-limiting enzyme in the synthesis of cholesterol. Mevalonate pathway involvement in HCC development is supported by accumulating data [114]. Additionally, the liver uses cholesterol to make bile acids that are hydroxylated steroids, which after being produced in the gut help to solubilize dietary cholesterol, lipids, fat-soluble vitamins, and other necessary nutrients. This facilitates the absorption of these nutrients into the liver. Primary bile acids such as cholic acid and chenodeoxycholic acid are produced by hepatocytes and can then be conjugated to glycine or taurine [115]. Glycocholic acid, which is cholic acid conjugated to glycine, is a secondary bile acid produced via the small intestine's microbiome [115,116]. Abnormal bile acid metabolism has been linked to HCC, and intrahepatic bile acid has been demonstrated to have a stimulatory impact on hepatic carcinogenesis [117].

Furthermore, blood samples from HCC patients showed abnormal phospholipid, FA, and bile metabolite metabolism [116]. Particularly, higher quantities of phosphatidylcholines (PC) were seen in early-stage and late-stage HCC patients in comparison to cirrhotic and normal individuals, suggesting a disruption in the metabolism of phospholipids [117].

Clinical implication of metabolites as potential biomarkers for HCC

Numerous metabolites of several metabolic pathways have been identified as possible biomarkers for HCC due to their alterations during HCC development and progression. These biomarkers might be used for early diagnosis and prediction of the development and progression of HCC. Furthermore, the breakthrough of multi-omics techniques allowed the discovery of novel metabolomics in body fluids and tissues, which could be used in clinical practice for HCC patients [118].

The following table (Table 1) summarizes the potential metabolomics and metabolites that could be used for early diagnosis and discrimination of HCC from chronic hepatitis and liver cirrhosis.

**Table 1:** Summary of the potential metabolomics and metabolites that could be used for early diagnosis and discrimination of HCC from chronic hepatitis and liver cirrhosis

Technique	Samples	Experimental group	Control group	Increased metabolites	Decreased metabolites	Outcome of the study	References
Capillary electrophoresis-time of flight-mass spectrometry (CE-TOF/MS)	Serum	Diethylnitrosamine-induced hepatocellular carcinoma (HCC) (n=7)	Healthy control (n=7)	Creatine	Betaine	Creatine/betaine ratio reflected the balance of methylation state could discriminate between cirrhotic patients and HCC area under the curve (AUC) of 0.928	[119]
H nuclear magnetic resonance (^1^H-NMR)	Urine	DEN-induced HCC (n=18)	Healthy control (n=18)	Creatinine, putrescine, choline, taurine	Hippurate	Hippurate, creatinine, putrescine, choline, and taurine levels could discriminate between controls from diethyl nitrosamine (DEN)-induced HCC animal models (AUC OF 0.812, 0.701, 0.738, 0.722, and 0.722)	[120]
	Serum						
Gas chromatography-time of flight-mass spectrometry (GC-TOFMS)	Serum	HCC serum (n=39)	Healthy control (n=61), hepatitis B (n=49), cirrhosis (n=52)	Glutamate	Palmitic acid, asparagine	Asparagine and β-glutamate could discriminate between liver cirrhosis and HCC (AUC of 0.906)	[121]
LC-MS liquid chromatography-mass spectrometry	Serum	HCC serum (n=30)	Hepatitis B (n=30), liver cirrhosis (n=29)	Taurodeoxy cholic acid, 1,2-diacyl-3-β-D-galactosyl-sn-glycerol	Glycyrrhizic acid	Taurodeoxy cholic acid (TCA) and 1,2-diacyl-3-β-d-galactosyl-sn-glycerol levels could differentiate liver cirrhosis from HCC (AUC: 0.747 and 0. 713) whereas 5-hydroxy-6E,8Z,11Z,14Z,17Z-eicosapentaenoic acid and glycyrrhizic acid levels could differentiate chronic hepatitis B from HCC (AUC: 0.921, 0.847)	[122]
^1^H-NMR	Serum	HCC serum (n=40)	Hepatitis C (n=22)	Choline, valine	Creatinine	Choline, valine and creatinine could differentiate between HCC and HCV patients with AUC of 0.83	[123]
GC/MS gas chromatography-mass spectrometry	Plasma	HCC plasma (n=22)	HCV-related cirrhosis (n=22)	Oleic acid, octanoic acid, glycine	Capric acid	Octanoic (caprylic) acid, oxalic acid, decanoic (capric) acid, oleic acid, and glycine could differentiate between HCC and liver cirrhosis (AUC: 0.937, 0.762, 0.846, 1, and 0.591, respectively)	[124]
Liquid chromatography-mass spectrometry, gas chromatography-mass spectrometry (LC-MS, GC-MS)	Serum	Early HCC serum (n=50)	Healthy control (n=50), liver cirrhosis (n=47)	Methionine, proline, ornithine	Pimelylcarnitine, octanoylcarnitine	A panel of methionine, proline, ornithine, pimelylcarnitine, and octanoylcarnitine differentiate HCC from liver cirrhosis AUC of 0.82	[125]
Gas chromatography-mass spectrometry (GC/MS)	Urine	HCC urine (n=20)	Healthy control (n=20)	Octanedioic acid, glycine, L-tyrosine, L-threonine, butanedioic acid	Hypoxanthine, pyrimidine	A diagnostic model was constructed with a combination of 18 marker metabolites or together with alpha-fetoprotein (AFP) were shown to be significantly different between the HCC and control groups with AUC = 0.9275.	[13]
Ultra-performance liquid chromatography-mass spectrometry (UPLC-MS)	Tissues	HCC tissues (n=30)	Distal non-cancerous tissues (n=30)	Succinyladenosine, uridine	Chenodeoxycholic acid, glycocholic acid	The combination of CDCA, LPC20:5, succinyladenosine, and uridine can be used as a biomarker panel to improve HCC sensitivity and specificity, discriminating HCC from liver cirrhosis with an AUC = 0.938, sensitivity of 93.3%, and specificity of 86.7%.	[126]
^1^H-NMR H nuclear magnetic resonance	Tissues	HCC with severe fibrosis (n=26)	HCC with mild fibrosis (n=26)	Glucose, phosphoethanolamine, triacylglyceride	Monounsaturated fatty acid	Differential expression levels of glucose, choline derivatives and phosphoethanolamine, monounsaturated FA, triacylglycerides were identified as specific signatures for identification of fibrosis level in HCC	[127]
Ultra-performance liquid chromatography-mass spectrometry (UPLC-MS)	Tissues	HCC with diabetes (n=34)	HCC without diabetes (n=26)	2-hydroxystearate	-	2-hydroxystearate was only overexpressed in the tumor tissues of HCC with diabetes and associated with the glucose level; other metabolites that are differentially expressed in the matched normal liver tissues of diabetes HCC compared to non-diabetes HCC patients	[128]

## Conclusions

In system biology techniques, metabolomics, a novel technology for biomarker discovery, assists in understanding the metabolic processes of viruses within cells and their relationships with one another. Metabolomics profiles could be a viable replacement for invasive diagnostic and prognostic methods because they can distinguish between various disease states. Alterations in the metabolism of organic acids, amino acids, glucose, and ACs are closely linked to the pathophysiology of HCC. Therefore, these metabolites may have a clinical impact on the diagnosis of HCC patients, potentially be used in follow-up, and may be useful in treatment monitoring. This study provides a targeted understanding of the altered metabolites in HCC and may be crucial in developing a metabolomics signature and biomarkers for the early diagnosis of HCC. Ultimately, the study of metabolomics has profoundly transformed the field of cancer research more than any other technique. 
